# Huntingtin-Interacting Protein 1 Promotes Vpr-Induced G2 Arrest and HIV-1 Infection in Macrophages

**DOI:** 10.3390/v13112308

**Published:** 2021-11-19

**Authors:** Tomoyuki Murakami, Ryosuke Matsuura, Nopporn Chutiwitoonchai, Masami Takei, Yoko Aida

**Affiliations:** 1 Viral Infectious Diseases Unit, RIKEN, 2-1 Hirosawa, Wako, Saitama 351-0198, Japan; tmurakam@umich.edu (T.M.); matsuura-ryosuke@g.ecc.u-tokyo.ac.jp (R.M.); nponopr@gmail.com (N.C.); 2 Laboratory of Global Infectious Diseases Control Science, Graduate School of Agricultural and Life Sciences, The University of Tokyo, 1-1-1 Yayoi, Bunkyo-ku, Tokyo 113-8657, Japan; 3 Division of Hematology and Rheumatology, Department of Medicine, Nihon University School of Medicine, 30-1 Oyaguchi, Kami-cho, Itabashi, Tokyo 173-8610, Japan; takei.masami@nihon-u.ac.jp

**Keywords:** HIV-1, Vpr, G2 arrest, HIP1, macrophage

## Abstract

Human immunodeficiency virus type 1 (HIV-1) modulates the host cell cycle. The HIV-1 accessory protein Vpr arrests the cell cycle at the G2 phase in dividing cells, and the ability of Vpr to induce G2 arrest is well conserved among primate lentiviruses. Additionally, Vpr-mediated G2 arrest likely correlates with enhanced HIV-1 infection in monocyte-derived macrophages. Here, we screened small-interfering RNA to reveal candidates that suppress Vpr-induced G2 arrest and identified Huntingtin-interacting protein 1 (HIP1) required for efficient G2 arrest. Interestingly, HIP1 was not essential for Vpr-induced DNA double-strand breaks, which are required for activation of the DNA-damage checkpoint and G2 arrest. Furthermore, HIP1 knockdown suppressed HIV-1 infection in monocyte-derived macrophages. This study identifies HIP1 as a factor promoting Vpr-induced G2 arrest and HIV-1 infection in macrophages.

## 1. Introduction

Vpr is an accessory gene product of the human immunodeficiency virus type 1 (HIV-1) and a small 15-kDa protein with multiple biological functions, including splicing regulation [1,2,3], support of virus release [4], nuclear import of the viral preintegration complex in macrophages [5,6,7], enhanced expression and processing of the envelope glycoprotein in macrophages [8,9,10], sustaining interleukin 6 expression to enhance HIV-1 replication [11], antagonism of exonuclease 1- and helicase-like transcription factor-mediated restriction in T cells through degradation of these proteins [12,13,14,15], regulation of apoptosis in both a positive and negative manner, and the induction of cell cycle arrest at the G2 phase in dividing cells [16,17,18,19,20,21]. Multiple functions of Vpr are exerted through interactions with various host factors, such as DNA damage-binding protein 1 (DDB1)- and cullin 4 (CUL4)-associated factor 1 (DCAF1), spliceosome-associated protein 145, p300, synthetic lethal of unknown (X) function 4 (SLX4), protein arginine N-methyltransferase 5, importin α, mini-chromosome maintenance protein10, and coiled-coil domain-containing-137 (CCDC137) [2,6,7,13,20,22,23,24,25,26,27]. The induction of G2 arrest is likely an important function for efficient viral replication because the ability of Vpr to cause cell cycle blockade is well conserved among primate lentiviruses [28,29]. Additionally, Vpr accelerates acute HIV-1 infection by exploiting proliferating CD4^+^ T cells, including regulatory CD4^+^ T cells, through G2 arrest and apoptosis in vivo [30].

Vpr induces DNA damage and activates ataxia telangiectasia-mutated and Rad3-related protein (ATR) to induce G2 arrest [31,32,33]. Vpr-induced G2 arrest requires the association of Vpr with the CUL4 ubiquitin ligase in association with DDB1 and DCAF1 [24,34,35,36,37,38]. In addition, recently, it has been shown that Vpr degrades CCDC137 to induce G2 arrest [22]. Although previous reports indicate that the interaction between CUL4–Vpr and the SLX4 complex is necessary for efficient induction of G2 arrest [25,39], Fregoso et al. [40] demonstrated that Vpr induces G2 arrest independent of SLX4. Thus, the full molecular mechanism(s) underlying Vpr-induced G2 arrest remain unknown.

Macrophages are a cellular target of HIV-1. In HIV-1-infected patients, macrophages reportedly act as a viral reservoir widely distributed throughout multiple tissues under the combination antiretroviral therapy [41,42]. Vpr enhances HIV-1 infection in macrophages through various mechanisms [6,7,8,9,10,11,12,43], and a Vpr mutant showing defective induction of a G2 arrest also fails to promote HIV-1 infection in macrophages [43]. Therefore, it is possible that Vpr-mediated G2 arrest is implicated in efficient HIV-1 infection in macrophages, and that understanding of Vpr-specific G2-arrest mechanism(s) could reveal how Vpr facilitates HIV-1 infection in macrophages.

Huntingtin-interacting protein 1 (HIP1) interacts with the protein encoded by the gene mutated in Huntington’s disease [44,45], an inherited neurodegenerative disorder caused by expansion of the codon CAG in the *Huntingtin* gene and resulting in the translation of a polyglutamine tract in the protein. The affinity of the Huntingtin–HIP1 interaction is inversely correlated with the polyglutamine-repeat length [44,45]. Additionally, HIP1 is associated not only with Huntingtin’s disease but also various cellular processes, including clathrin-mediated endocytosis [46,47,48], tumorigenesis [49,50], and neuronal cell death [51,52]. 

In this study, we investigated the novel molecular mechanism of Vpr-mediated G2 arrest by screening candidate(s) directly involved in this process through the use of a small-interfering (si)RNA mini-library of target genes and CELAVIEW RS100, an imaging-based screening microscope. CELAVIEW RS100 automatically acquires cellular fluorescence images and quantitatively analyzes the morphology and fluorescence signal in a large number of cells. Compared with flow cytometry analysis, CELAVIEW RS100 enables high-throughput analysis of DNA contents in the Hoechst33342-stained nuclei of large numbers of Vpr-expressing cells. As a second screening, we determined whether the candidate(s) were involved in Vpr-induced G2 arrest using flow cytometry analysis. Screening results and a further validation experiment identified HIP1 as a novel host factor involved in Vpr-induced G2 arrest, after which we examined the effect of *HIP1* knockdown on HIV-1 infection in macrophages.

## 2. Materials and Methods

### 2.1. Cell Culture and Transfection

HeLa cells and 293T cells were grown in Dulbecco’s modified Eagle’s medium (GIBCO, Gaithersburg, MD, USA) supplemented with 10% heat-inactivated fetal bovine serum (FCS; Sigma-Aldrich, St. Louis, MO, USA). Human PBMCs were isolated on a Ficoll (Lymphosepal; Immuno-Biological Laboratories, Minneapolis, MN, USA) gradient from a healthy donor. Monocytes were selected from PBMCs using CD14 MicroBeads (Miltenyi Biotec, Bergisch Gladbach, Germany) and a MACS separation column (Miltenyi Biotec) with a Quandro MACS separation unit (Miltenyi Biotec) according to manufacturer instructions. Monocytes were cultured at the desired density in 6- or 24-well plates and grown in Roswell Park Memorial Institute (RPMI)-1640 medium (Invitrogen, Carlsbad, CA, USA) containing 10% heat-inactivated FCS (Culture Biosciences, San Francisco, CA, USA), 5% AB serum (Sigma-Aldrich), and a 10 ng/mL macrophage colony-stimulating factor (M-CSF; PeproTech, Rocky Hill, NJ, USA) for 1 week until they spontaneously differentiated into mature macrophages. 

Plasmid transfection was performed using FuGENE HD (Promega, Madison, WI, USA) or Lipofectamine2000 (Invitrogen). siRNA transfection was performed using Lipofectamine2000 or Lipofectamine RNAi MAX (Invitrogen). siRNA and plasmid co-transfection was performed using Lipofectamine2000. siRNA transfection for macrophages was performed using Lipofectamine RNAi MAX. Macrophages were transfected with 50 nM siRNA in Opti-MEM (GIBCO). At 4-h post-transfection, macrophages were washed and cultured in an RPMI-1640 medium containing 10% heat-inactivated FCS, 5% AB serum, and 10 ng/mL M-CSF for 20 h. Cells were subjected to subsequent rounds of transfection, washing, and culturing as described. 

### 2.2. Plasmid Construction

The expression vector pME18Neo encoding N-terminal Flag-tagged WT Vpr (pME/F-Vpr) and pGEX-6P-3 encoding N-terminal GST-tagged Vpr (pGEX-6P-3/GST-Vpr) have been described previously [53,54]. The molecular clone vectors pNL4-3-Luc-*env*(−) and pNL4-3-Luc-*env*(−)*vpr*(−) and an expression construct for the vesicular stomatitis virus G protein (VSV-G) (pVSV-G) were kindly gifted by Dr. Ishizaka (Department of Intractable Diseases, National Center for Global Health and Medicine). For construction of the vector pME/F-Vpr-IRES-ZsGreen1 and the control vector pME/F-IRES-ZsGreen1, a fragment containing an IRES sequence and a ZsGreen1-coding sequence was amplified by a polymerase chain reaction (PCR) using the primers 5′-CCCAAACTTAAGCTTGGTACCGA-3′ and 5′-TAGCGGCCGCTCAGGGCAAGGCGGAGCCGGAG-3′ and pRetroX-IRES-ZsGreen1 (Clontech Laboratories, Mountain View, CA, USA) as a template. The PCR fragment was subcloned into pME/F-Vpr and pME18Neo/Flag at the *Not*Ι site. 

For construction of the expression vector pCAGGS encoding N-terminal HA-tagged HIP1 (pCAGGS/HA-HIP1), human *HIP1* mRNA was amplified by reverse transcription (RT)-PCR from RNA derived from HeLa cells. RT was performed with an oligo-dT primer, and PCR was performed using the primers 5′-AAAGATATCGGATCGGATGGCCAGCTCCATGAAGCAGGTGCCCAA-3′ and 5′-AAAGCGGCCGCCTATTCTTTTTCGGTTACCACTTC-3′. The PCR fragment was subcloned into the pCAGGS/HA vector between the *Eco*RV and *Not*I sites. For construction of the pCAGGS/HA-siR-HIP1 vector, the mutant was generated using standard PCR mutagenesis techniques using pCAGGS/HA-HIP1 as a template.

### 2.3. siRNA

siRNAs against 256 genes were prepared as an siRNA mini-library [55], and the identities are shown in Kameoka et al. [55]. siRNAs targeting *HIP1* and *DCAF1* were designed using BLOCK-iT RNAi Designer (Invitrogen). The siRNA forward sequences targeting *HIP1* were 5′-CACAGACCUUCUGGUCUGUUGUCAA-3′ for siRNA#1 and 5′-GGAGCUAAUGGUGUGUUCUCAUGAA-3′ for siRNA#2. The siRNA forward sequence targeting *DCAF1* was 5′-CCCUGGGUGAUUGGCACCAAUUAUA-3′.

### 2.4. Western Blotting

Cells were lysed for 30 min on ice in 10 mM Tris-HCl (pH 8.0), 150 mM NaCl, 5 mM EDTA, 1% Triton X-100, and 0.1% SDS supplemented with a protease-inhibitor cocktail (Roche). Lysates were mixed with an SDS-PAGE sample buffer and boiled for 5 min. Protein concentrations were determined with a BCA protein assay kit (Pierce; Thermo Fisher Scientific, Waltham, MA, USA) using bovine serum albumin as a standard. Equal amounts of total protein were examined by Western blotting using the following antibodies: anti-Flag monoclonal antibody (mAb; M2; Sigma-Aldrich), anti-Flag polyclonal antibody (Sigma-Aldrich), anti-HA polyclonal antibody (Sigma-Aldrich), anti-β-actin mAb (Sigma-Aldrich), anti-HA mAb (MBL International, Woburn, MA, USA), anti-HIP1 mAb (Novus Biologicals, Littleton, CO, USA), anti-VprBP polyclonal Ab (Proteintech, Rosemont, IL, USA), horseradish-peroxidase (HRP)-conjugated goat anti-mouse IgG (Amersham Biosciences, Little Chalfont, UK), and HRP-conjugated goat anti-rabbit IgG (Amersham Biosciences). Signals were visualized after treatment with SuperSignal West Pico chemiluminescent substrate (Pierce; Thermo Fisher Scientific).

### 2.5. Immunofluorescent Staining

HeLa cells on a cover slip were co-transfected with siRNAs and either pME/F-Vpr-IRES-ZsGreen1 or pME/F-IRES-ZsGreen1 and cultured for 48 h. Cells were fixed in 3.6% formaldehyde in a phosphate-buffered saline (PBS) for 10 min at room temperature (RT) and permeabilized with PBS containing 0.2% Triton X-100 for 5 min on ice. After treating with the PBS containing 5% skim milk for 15 min at RT, the cells were stained with the anti-γ-H2AX mAb (Sigma-Aldrich), followed by Alexa Fluor 594-conjugated secondary antibody (Invitrogen) and 5 μg/mL Hoechst33342 in PBS containing 2.5% skim milk for 30 min. After rinsing with PBS, the cover slip was mounted on glass slides in the PBS containing 90% glycerol and analyzed by confocal laser scanning microscopy (Olympus, FV1000D).

### 2.6. Analysis of Cell Cycle Profiles by CELAVIEW RS100

HeLa cells were plated in 24-well polystyrene plates. To analyze the effect of siRNA transfection on Vpr-induced G2 arrest, the cells were co-transfected with siRNAs and either pME/F-Vpr-IRES-ZsGreen1 or pME/F-IRES-ZsGreen1 and cultured for 48 h. To analyze the effect of *HIP1* overexpression on the inhibition of Vpr-induced G2 arrest following *HIP1* knockdown, the cells were co-transfected with siRNAs, pME/F-Vpr-IRES-ZsGreen1, and pCAGGS/HA-siR-HIP1 or pCAGGS/HA and cultured for 48 h. These cells were fixed and stained with 3.6% formaldehyde containing 5 μg/mL Hoechst33342 for 10 min at room temperature and then washed 3 times with PBS. For each sample, at least 200 ZsGreen1-positive cells were observed and analyzed using a CELAVIEW microscope (RS100; Olympus, Tokyo, Japan).

### 2.7. Analysis of Cell Cycle Profiles by Flow Cytometry

HeLa cells were co-transfected with siRNAs and either pME/F-Vpr-IRES-ZsGreen1 or pME/F-IRES-ZsGreen1 as a control and cultured for 48 h. The cells were harvested and fixed with 1% formaldehyde, followed by 70% ethanol. Fixed cells were incubated in PBS containing RNase A (50 μg/mL) at 37 °C for 20 min and then stained with propidium iodide (40 μg/mL). For each sample, at least 7000 cells were analyzed using a FACS Calibur instrument (Becton Dickinson, Franklin Lakes, NJ, USA) with CELL Quest software (Becton Dickinson). Ratios of the numbers of cells in G1 and G2/M phases (G2/M:G1 ratios) were calculated using ModFit LT software (Verity Software House, Topsham, ME, USA).

### 2.8. Immunoprecipitation

293T cells were co-transfected with the indicated vectors, and at 48-h post-transfection, the cells were lysed with a lysis buffer [50 mM Tris-HCl (pH 7.4), 150 mM NaCl, and 0.5% NP-40] supplemented with a protease-inhibitor cocktail (Roche, Basel, Switzerland) for 30 min on ice. The lysates were centrifuged at 15,000 rpm for 5 min, and the supernatants were collected and mixed with Anti-HA agarose beads (Sigma-Aldrich) and incubated at 4 °C for 18 h with gentle rotation. The beads were washed 5 times with a lysis buffer, and the bound proteins were eluted using the HA peptide (Sigma-Aldrich). Eluted proteins were fractionated by 6% and 15% sodium dodecyl sulfate polyacrylamide gel electrophoresis (SDS-PAGE) and analyzed Western blot.

### 2.9. Protein Expression and Purification

Recombinant glutathione-S-transferase (GST) or GST-Vpr was expressed in *Escherichia coli* BL21 CodonPlus (DE3)-RIL cells (Stratagene, San Diego, CA, USA). Expression was induced with 1 mM isopropyl β-D-1-thiogalactopyranoside at 16 °C for 24 h, followed by lysis with a BugBuster reagent (Novagen; Merck, Kenilworth, NJ, USA) according to manufacturer instructions. The lysate was cleared by centrifugation, and the soluble fraction was mixed with glutathione-Sepharose 4 FastFlow beads (GE Healthcare, Pittsburgh, PA, USA), which were centrifuged and washed with a BugBuster reagent and phosphate-buffered saline (PBS).

To express and purify HA-HIP1, 293T cells were transfected with pCAGGS/HA-HIP1 using a FuGene HD transfection reagent (Promega), and at 48-h post-transfection, the cells were collected and lysed with a wash buffer. The lysates were centrifuged at 15,000 rpm for 5 min, and the supernatants were collected, mixed with Anti-HA agarose beads (Sigma-Aldrich), and incubated at 4 °C for 1 h with gentle rotation. The affinity beads were washed with a wash buffer twice, and the HA-HIP1 was eluted using the HA peptide (Sigma-Aldrich).

### 2.10. Pull-Down Assay

Purified HA-HIP1 was incubated with GST or GST-Vpr preadsorbed onto glutathione-Sepharose 4 FastFlow beads at 4 °C for 2 h in a wash buffer. The beads were then washed with the wash buffer 5 times, and bound proteins were eluted by incubation with a sample buffer for SDS-PAGE at 100 °C for 5 min. Eluted proteins were fractionated by 6% SDS-PAGE for Western blotting.

### 2.11. Viral Stock and Viral Infection of Macrophages

To generate viral stocks, 293T cells were co-transfected with pNL4-3-Luc-*env*(−) or pNL4-3-Luc-*env*(−)*vpr*(−) and pVSV-G using FuGENE HD (Promega), and the virus was harvested at 48-h post-transfection. HIV-1 titers were measured using an anti-p24 enzyme-linked immunosorbent assay kit (Ryukyu Immunology, Okinawa, Japan).

Primary macrophages in 24-well plates were inoculated with VSV-G pseudotyped reporter viruses, NL-Luc-E^−^R^+^ (VSV-G) or NL-Luc-E^−^R^−^ (VSV-G); 4 ng of p24 antigen, cultured for 6 days, harvested, and lysed in a luciferase assay substrate (Promega). Infectivity was determined by the measurement of luciferase activity. 

### 2.12. Statistical Analysis

Statistical analyses were performed by Prism 8.0 (GraphPad software, San Diego, CA, USA). For two-group comparisons, a two-tailed Student′s *t*-test was used. Data are presented as mean ± SD and were considered statistically significant when the *p* value was <0.05. 

## 3. Results

### 3.1. HIP1 Enhances Vpr-Induced G2 Arrest

**Figure 1:**
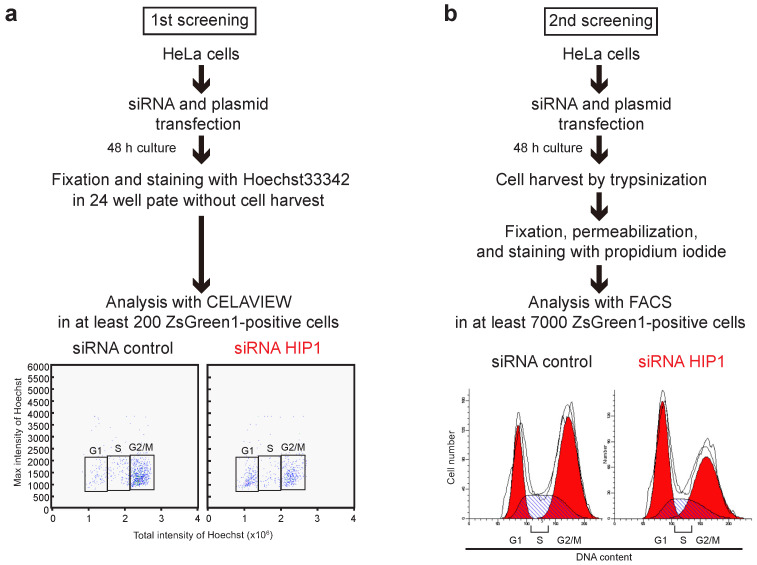
Summary of siRNA screening for candidate suppressors of Vpr-induced G2 arrest. (**a**) For the initial screening, HeLa cells were co-transfected with pME/F-Vpr-IRES-ZsGreen1 and 100 nM individual siRNAs from the siRNA mini-library or control siRNA. At 48-h post-transfection, cells were fixed and stained with 5 μg/mL Hoechst33342 to measure DNA content in at least 200 ZsGreen1+ cells using CELAVIEW RS100. (**b**) For secondary screening, HeLa cells were co-transfected with pME/F-Vpr-IRES-ZsGreen1 and control siRNA or 100 nM of individual siRNAs that inhibited Vpr-induced G2 arrest according to the initial screen. At 48-h post-transfection, cells were harvested by trypsinization, fixed, permeabilized, treated with RNase A, and stained with 50 μg/mL propidium iodide to measure DNA content in at least 7000 ZsGreen1+ cells by flow cytometry. The traces on the cell cycle histograms are written by the ModFit LT software automatically.

To identify new cellular factor(s) involved in the Vpr-induced G2 arrest, we used CELAVIEW RS100 for the initial screening in combination with an siRNA mini-library containing 256 siRNAs [55]. The siRNAs in this mini-library target functional genes that are involved in intracellular signal transduction pathways, intracellular transportation processes, and the cytoskeletal system [55]. CELAVIEW RS100 enables high-throughput analysis of DNA contents via automated image acquisition and data analysis. As shown in Figure 1a, HeLa cells were transfected with a bicistronic vector pME18Neo/Flag-Vpr-internal ribosomal entry site (IRES)-ZsGreen1 (pME/F-Vpr-IRES-ZsGreen1) encoding Flag tagged-wild-type (WT) Vpr (F-Vpr) and ZsGreen1 as a marker of Vpr expression, together with the siRNAs. At 48-h post-transfection, the cells were fixed and stained with Hoechst33342 for analysis of DNA contents using CELAVIEW RS100. Among the 256 siRNAs, 36 inhibited Vpr-induced G2 arrest in ZsGreen1+ cells (Figure 1a). 

To confirm the screening results, we performed secondary screening to investigate the effect of these siRNAs on the cell cycle progression in HeLa cells using flow cytometry analysis (Figure 1b). All siRNAs that we tested showed an inhibitory effect to Vpr-induced G2 arrest. 

**Figure 2:**
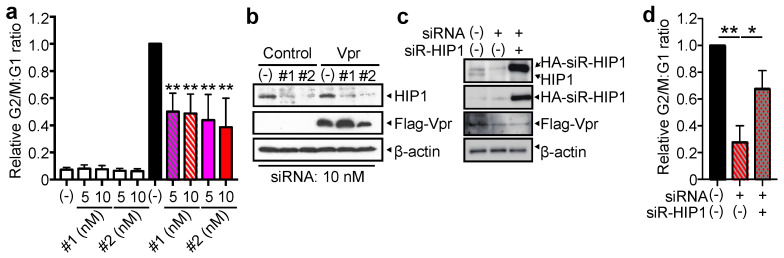
HIP1 enhances Vpr-induced G2 arrest. (**a**,**b**) HeLa cells were co-transfected with pME/F-Vpr-IRES-ZsGreen1 or control pME/F-IRES-ZsGreen1 and 5 or 10 nM either *HIP1* (siRNA#1 and #2) or control siRNA and cultured for 48 h. (**a**) Cells were fixed and stained with 5 μg/mL Hoechst33342 to measure DNA content in ZsGreen1+ cells using CELAVIEW RS100. The relative G2/M:G1 ratio was plotted, and data represent the mean ± SD of three independent experiments. (**b**) Cells were lysed and subjected to 6% and 15% SDS-PAGE and Western blot using an anti-HIP1 mAb, anti-Flag M2 mAb, and anti-β-actin mAb. (**c**,**d**) HeLa cells were co-transfected with pME/F-Vpr-IRES-ZsGreen1 and pCAGGS/HA-siR-HIP1 (carrying synonymous nucleotide mutations at the third codon of the siRNA-targeting site) or control pCAGGS/HA and either 10 nM HIP1 (siRNA#2) or control siRNA and then cultured for 48 h. (**c**) Cells were lysed and subjected to 6% and 15% SDS-PAGE and Western blot using an anti-HIP1 mAb, anti-Flag M2 mAb, anti-HA mAb, and anti-β-actin mAb. (**d**) Cells were fixed, permeabilized, stained with the anti-HA mAb, and then with Alexa Fluor 594-conjugated secondary Ab and Hoechst33342. The DNA content of ZsGreen1+ and Alexa Fluor 594+ cells was analyzed by CELAVIEW RS100. The relative G2/M:G1 ratio was plotted, and data represent the mean ± SD of three independent experiments. * *p* < 0.05, ** *p* < 0.01 via two-tailed Student’s *t*-test.

To validate whether these target genes truly play a role in G2 arrest, we designed siRNAs targeting different protein-coding sequences and analyzed their effects on the cell cycle profile of Vpr-expressing cells using CELAVIEW RS100. We found that among the siRNAs, only siRNAs targeting *HIP1* (#1 and #2), which is predominantly expressed in the brain and interacts with Huntingtin [44,45], inhibited Vpr-induced G2 arrest (Figure 2a). In addition, these siRNAs did not affect cell cycle progression in cells transfected with pME18Neo/Flag-IRES-ZsGreen1 (pME/F-IRES-ZsGreen1) and either siRNA (Figure 2a). 

These results suggest that most siRNAs in the library possibly attenuate Vpr-induced G2 arrest through an off-target effect and that HIP1 could contribute to Vpr-induced G2 arrest. Subsequently, we focused on the role of HIP1 in Vpr-induced G2 arrest. Western blotting confirmed the knockdown of HIP1 levels in HeLa cells transfected with either pME/F-IRES-ZsGreen1 or pME/F-Vpr-IRES-ZsGreen1 together with either newly designed siRNA#1 or #2 (Figure 2b). Although transfection with siRNA#2 reduced the level of Vpr, siRNA#1 did not affect Vpr expression (Figure 2b). Therefore, these results suggested that since both siRNA#1 and #2 similarly inhibited Vpr-induced G2 arrest (Figure 2a), *HIP1* knockdown can suppress Vpr-induced G2 arrest without reducing the level of Vpr expression. To examine whether the overexpression of siRNA-resistant *HIP1* (siR-HIP1), which is not knocked down by siRNA#2 due to its synonymous nucleotide mutations in the third codon of the siRNA#2-targeted sequence, restores the ability of Vpr to induce G2 arrest, HeLa cells were co-transfected with pME/F-Vpr-IRES-ZsGreen1, either siRNA#2 or control siRNA, and either an siR-HIP1 expression vector or empty vector. We thus observed the ectopic expression of siR-HIP1 even when HeLa cells were co-transfected with siRNA#2 (Figure 2c), with the overexpression of siR-HIP1 restoring G2 arrest by up to ~40% (Figure 2d). These results suggested that HIP1 augments Vpr-induced G2 arrest.

### 3.2. HIP1 Interacts with Vpr

**Figure 3:**
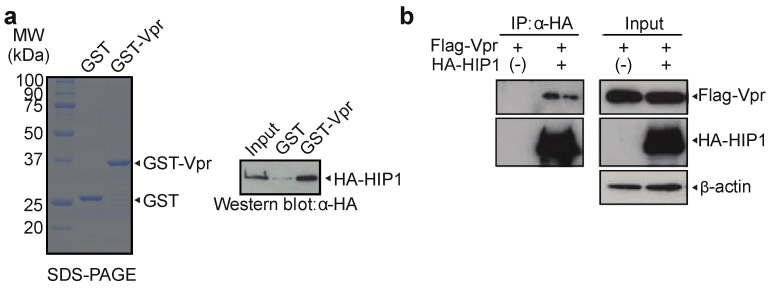
Vpr interacts with HIP1. (**a**) GST and GST-Vpr were resolved by 12% SDS-PAGE and stained with Coomassie Brilliant Blue (CBB) (left). Glutathione-Sepharose beads combined with the GST-Vpr or GST alone were incubated with purified HA-HIP1 protein. The bound fractions and 10% of the input were analyzed by Western blot using the anti-HA mAb (right). The positions of HA-HIP1, GST-Vpr, and GST are indicated. (**b**) 293T cells were co-transfected with pME/FVpr and pCAGGS/HA-HIP1 or control pCAGGS/HA. At 48-h post-transfection, cells were lysed, and the lysates were subjected to immunoprecipitation assays using anti-HA agarose and the HA peptide. The bound fractions and inputs were analyzed by Western blotting using the anti-HA polyclonal Ab, anti-Flag polyclonal Ab, and anti-β-actin mAb.

To investigate whether HIP1 interacts with Vpr, we used a GST pull-down assay. Incubating recombinant GST-tagged Vpr (GST-Vpr; immobilized on glutathione-Sepharose beads) with an HA-tagged HIP1 protein (HA-HIP1) purified from 293T cells transfected with pCAGGS/HA-HIP1, followed by GST pull-down assays, revealed that HIP1 bound Vpr (Figure 3a). To confirm the interaction between HIP1 and Vpr in cells, we performed immunoprecipitation assays using lysate from 293T cells transfected with pME18Neo/Flag-Vpr together with pCAGGS/HA-HIP1, which revealed an HIP1–Vpr interaction (Figure 3b). These results suggested that Vpr possibly induces G2 arrest via interactions with HIP1.

### 3.3. HIP1 Is Dispensable for Vpr-Induced DNA Double-Strand Breaks

**Figure 4:**
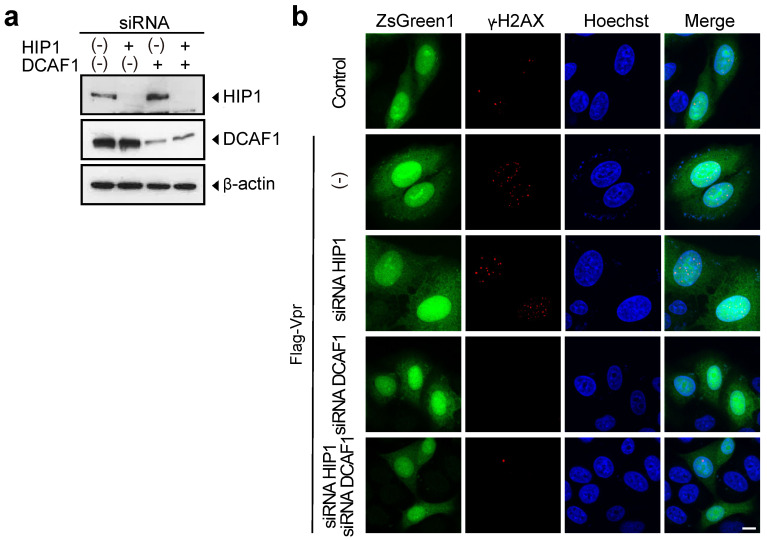
Vpr induces DNA double-strand breaks in *HIP1*-knockdown cells. (**a**) HeLa cells were transfected with 5 nM *HIP1* (siRNA#2), *DCAF1*, or a combination of 5 nM *HIP1* (siRNA#2) and 5 nM *DCAF1* siRNAs. The total amount of siRNA was adjusted to 10 nM with control siRNA. At 48-h post-transfection, cells were lysed and subjected to SDS-PAGE and Western blot using the anti-HIP1 mAb, anti-DCAF1 mAb, and anti-β-actin mAb. The positions of HIP1, DCAF1, and β-actin are indicated. (**b**) HeLa cells were co-transfected with pME/F-Vpr-IRES-ZsGreen1 or control pME/F-IRES-ZsGreen1 and 5 nM *HIP1* (siRNA#2), *DCAF1*, or a combination of 5 nM *HIP1* (siRNA#2) and/or 5 nM *DCAF1* siRNAs. The total amount of siRNA was adjusted to 10 nM with control siRNA. At 48-h post-transfection, cells were fixed, permeabilized, and stained with the anti-γ-H2AX mAb, followed by Alexa Fluor 594-conjugated secondary antibody and 5 μg/mL Hoechst33342. Cells were analyzed by a confocal microscopy. Cells showing green fluorescence (ZsGreen1+) and red foci indicated the presence of DNA double-strand breaks, and blue fluorescence indicated nuclei. The scale bar represents 10 μm.

Vpr induces DNA double-strand breaks and G2 arrest via interactions with the CUL4 ubiquitin ligase in association with DDB1 and DCAF1 [24,34,35,36,37,38,56]. To test whether HIP1 contributes to Vpr-induced DNA double-strand breaks, we analyzed the effect of *HIP1* knockdown on the Vpr-induced formation of DNA-repair foci containing phosphorylated histone 2A variant X (γ-H2AX), which forms distinct nuclear foci following Vpr-induced DNA double-strand breaks [32]. After transfection with pME/F-Vpr-IRES-ZsGreen1 together with *HIP1* siRNA#2 and/or *DCAF1* siRNA (as a positive control siRNA for inhibition of Vpr-induced DNA double-strand breaks [56]), we performed immunofluorescence staining and confocal microscopy analysis to detect γ-H2AX foci. Figure 4a shows Western blot confirmation of decreased HIP1 and DCAF1 levels. Interestingly, HIP1 knockdown alone did not suppress Vpr-induced formation of γ-H2AX foci (Figure 4b). By contrast, knockdown of DCAF1 alone or both HIP1 and DCAF1 completely inhibited Vpr-induced formation of γ-H2AX foci (Figure 4b). These results indicated that HIP1 is dispensable for Vpr-induced DNA double-strand breaks, and that HIP1 possibly plays a role in Vpr-induced G2 arrest independent of DCAF1. 

### 3.4. HIP1 Promotes HIV-1 Infection in Macrophages

**Figure 5:**
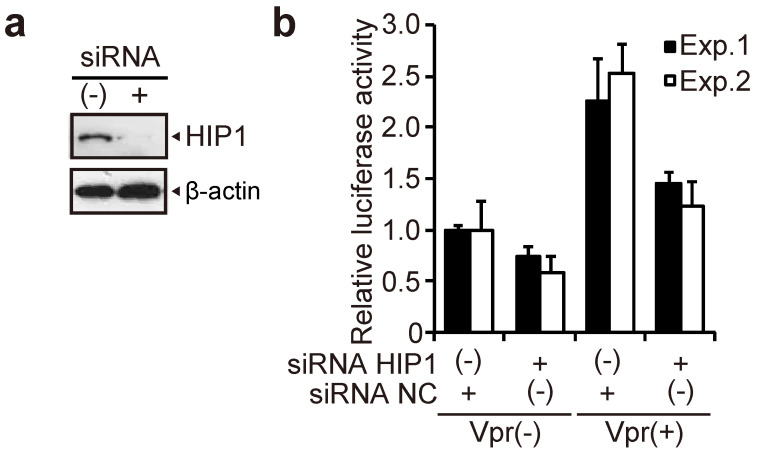
HIP1 enhances HIV-1 infection in macrophages in a Vpr-dependent manner. Monocytes isolated from PBMCs of human healthy donor were subsequently differentiated into macrophages by addition of M-CSF, followed by their transfection with 50 nM *HIP1* (siRNA#2; siRNA HIP1) and control siRNA (siRNA NC). (**a**) At 48-h post-transfection, cells were lysed and subjected to 6% SDS-PAGE and Western blotting using the anti-HIP1 mAb and anti-β-actin mAb. The positions of HIP1 and β-actin are indicated. (**b**) At 24-h post-transfection, cells were infected with 4 ng p24 VSV-G-pseudotyped-HIV-1 or -HIV-1 ΔVpr. At 6-days post-infection, cells were lysed, and infectivity was determined by measuring luciferase activity. Data represent the mean ± SD of triplicate wells.

In primary macrophages, Vpr enhances HIV-1 infection, whereas a Vpr mutant that is unable to induce G2 arrest does not enhance HIV-1 infection in macrophages [43]. Therefore, it is possible that HIP1 also contributes to efficient HIV-1 infection in macrophages. To investigate whether HIP1 is involved in enhancing Vpr-mediated HIV-1 infection in primary macrophages, monocyte-derived macrophages were differentiated from monocytes isolated from human peripheral blood mononuclear cells (PBMCs), as described previously [7,27]. These macrophages were transfected with *HIP1* siRNA#2 and then infected with VSV-G-pseudotyped NL4-3-Luc HIV-1 encoding either WT Vpr (Vpr+ virus) or truncated Vpr (Vpr^−^ virus), which can only support a single round of HIV-1 replication, at 24-h post-transfection. Following the confirmation of attenuated HIP1 levels by Western blotting (Figure 5a), we determined infectivity by measuring luciferase activity at 6-days post-infection. The luciferase activity in both Vpr+ and Vpr^−^ virus-infected cells was reduced by *HIP1* knockdown, with the reduction in Vpr+ virus-infected cells slightly larger than that in Vpr^−^ virus-infected cells (~43% in Vpr+ virus vs. ~33% in Vpr^−^ virus) (Figure 5b). These results suggested that although HIP1 contributes to HIV-1 infection in macrophages, it likely promotes stronger infection-related activity via cooperation with Vpr. 

## 4. Discussion

Vpr induces cell cycle arrest at the G2 phase and promotes HIV-1 infection, especially in macrophages [6,7,8,9,10,11,12,18]. The abilities of Vpr to induce G2 arrest and promote HIV-1 infection in macrophages probably correlate with each other. Indeed, Vpr causes G2 arrest and enhances HIV-1 gene expression in both primary CD4^+^ T cells and macrophages through the depletion of CCDC137 [22], and a Vpr-mutant that is defective in G2 arrest is not able to augment HIV-1 infection in macrophages [43]. Therefore, identifying the host genes with a role in Vpr-induced G2 arrest will possibly lead to an understanding of how Vpr enhances HIV-1 infection in macrophages. Various factors are required for Vpr-induced G2 arrest, including activation of the ATR pathway and interaction with CUL4 ubiquitin ligase in association with DDB1 and DCAF1 [24,25,31,32,33,34,36,37,38,57]. In the present study, siRNA screening identified HIP1 as a new host factor that modulates Vpr-induced G2 arrest. We demonstrated that HIP1 enhances Vpr-induced G2 arrest but is not required for Vpr-induced DNA double-strand breaks. Additionally, we found that HIP1 interacts with Vpr. Furthermore, we revealed that HIP1 contributes to efficient HIV-1 infection in macrophages.

Notably, we demonstrated that HIP1 is not necessary for Vpr-induced DNA double-strand breaks. However, it remains to be determined how HIP1 promotes Vpr-induced G2 arrest, and we thus propose three possibilities as follows: (i) HIP1 plays a role in a DNA damage response signal transduction pathway, (ii) HIP1 contributes to the inhibition of double-strand break repair mediated by Vpr, or (iii) HIP1 attenuates G1 arrest in Vpr-expressing cells. As for (i), after Vpr induces DNA double-strand breaks, the DNA damage response is activated to arrest the cell cycle at the G2 phase. The DNA damage response is a signal transduction pathway, which consists of various factors, such as ATR and checkpoint kinases [58]. Therefore, HIP1 might play a role in the signal transduction pathway but not in the induction of DNA damage. Related to this, Li et al. [59] reported that although protein phosphatase 2 (PP2A) is important for Vpr-induced G2 arrest, Vpr-induced DNA double-strand breaks are not inhibited by *PP2A* knockdown. Thus, HIP1 possibly collaborates with PP2A to enhance Vpr-induced G2 arrest independent of DNA damage induction. As for (ii), recently, it has been reported that in addition to the induction of DNA damage, Vpr suppresses DNA break repair to arrest the cell cycle at the G2 phase [60]. A mutant Vpr, R80A, which fails to induce G2 arrest, can induce DNA damage but not inhibit DNA repair [60]. Therefore, since HIP1 is dispensable for Vpr-induced double-strand breaks, HIP1 might be involved in the Vpr-mediated inhibition of DNA break repair. As for (iii), even when HIP1 is knocked down, Vpr induces DNA double-strand breaks, suggesting that the DNA damage response is active in Vpr-expressing cells without the expression of HIP1. Thus, the cell cycle of Vpr-expressing cells might be arrested at the G1 phase in the absence of HIP1. Previously, our group reported that a carboxy-terminally truncated mutant of Vpr, C81, induces G1 arrest but not G2 arrest [61]. Therefore, C81 might not be able to cooperate with HIP1 to induce G2 arrest.

We found that HIP1 interacts with Vpr. However, it is not clear whether Vpr requires interaction with HIP1 to induce G2 arrest and facilitate HIV-1 infection in macrophages. If the interaction is essential for efficient Vpr-induced G2 arrest and HIV-1 infection in macrophages, whether Vpr interacts with HIP1 directly or indirectly, whether the interaction between Vpr and HIP1 alters the structure of Vpr, whether HIP1 affects the binding of Vpr to other host proteins via the interaction, and which domains of Vpr and HIP1 mediate the interaction remain to be determined. HIP1 binds to actin through the talin-like domain [62]. Vpr also likely binds to cytoplasmic actin [63]. Therefore, the talin-like domain of HIP1 might be required for interactions with Vpr through actin in the cytoplasm. Even if the interaction between HIP1 and Vpr is not necessary for efficient Vpr-induced G2 arrest and HIV-1 infection in macrophages, the presence of HIP1 might affect the interaction between Vpr and other host proteins. Therefore, it would be interesting to know whether the knockdown of *HIP1* alters interactors of Vpr.

In the present study, we showed that compared to that with Vpr-deleted HIV-1, *HIP1* knockdown slightly but effectively inhibited WT HIV-1 infection in macrophages. This result suggested that HIV-1 replication in macrophages is enhanced by HIP1 in corporation with Vpr. In addition to that in macrophages, Vpr enhances HIV-1 infection and HIV-1 gene expression in CD4^+^ T cells [15,16,22,64,65]. Therefore, additional studies are needed to determine whether the knockdown/knockout of HIP1 could affect the Vpr-mediated enhancement of HIV-1 replication in CD4^+^ T cells.

In HIV-infected CD4^+^ T cells, Vpr induces G2 arrest [18], suggesting that the knockdown/knockout of HIP1 suppresses Vpr-induced G2 arrest in infected CD4^+^ T cells. However, the ability of Vpr to induce G2 arrest is dispensable for the cytopathic effects of Vpr in infected cells [66]. Thus, the knockdown/knockout of HIP1 in CD4^+^ T cells would not completely block HIV-1’s deleterious effects.

In summary, the results of the present study suggest that HIP1 augments Vpr-induced G2 arrest and HIV-1 infection in macrophages and that HIP1 interacts with Vpr. However, whether Vpr-induced G2 arrest and Vpr-mediated enhancement of HIV-1 infection requires the interaction between HIP1 and Vpr needs to be clarified. Although HIP1 slightly but efficiently enhanced WT HIV-1 infection in macrophages relative to that by Vpr-deleted HIV-1, the associated mechanism(s) remain to be elucidated. Future investigations are required to determine the exact roles of HIP1 in Vpr-induced G2 arrest and HIV-1 infection in macrophages, as well as to provide insights into correlations between the two functions of Vpr.

## Data Availability

The data presented in this study are available on request from the corresponding author.
